# How life-cycle real-world evidence can bridge evidentiary gaps in precision oncology

**DOI:** 10.3389/fmed.2025.1563950

**Published:** 2025-09-02

**Authors:** Emanuel Krebs, Deirdre Weymann, Tania Bubela, Dean A. Regier

**Affiliations:** ^1^Regulatory Science Lab, BC Cancer Research Institute, Vancouver, BC, Canada; ^2^Faculty of Health Sciences, Simon Fraser University, Burnaby, BC, Canada; ^3^Aga Khan University, Karachi, Pakistan; ^4^School of Population and Public Health, University of British Columbia, Vancouver, BC, Canada

**Keywords:** real-world evidence (RWE), real-world data (RWD), life-cycle assessment, causal inference, regulatory science, decision making, regulatory acceptance and use, precision oncology

## Abstract

Precision oncology uses omics-based diagnostic technologies to inform histology-agnostic cancer treatment. To date, health system implementation remains limited owing to high uncertainty in regulatory and reimbursement evidence submissions. In this perspective, we describe a life-cycle approach to the evaluation of precision oncology technologies that addresses evidentiary uncertainty and is grounded in real-world evidence (RWE) derived using data routinely collected by healthcare systems. We consider the role for RWE in international regulatory and reimbursement decision-making, review common biases for observational precision oncology evaluations, make specific recommendations for RWE study design and analysis, and specify healthcare system requirements for data collection. We then explore how decision-grade real-world data can support the generation of decision-grade RWE, ultimately enabling real-world life-cycle assessment for precision oncology.

## Introduction

Regulatory decisions aim to ensure that healthcare technologies are safe and effective, with reimbursement agencies in many jurisdictions adding an analysis of cost-effectiveness. Collectively, these decision processes manage uncertainty in the evidence base. Advances in precision oncology challenge regulatory and reimbursement decisions. Precision oncology refers to a suite of ‘omics-based diagnostics (e.g., genomics, transcriptomics, and metabolomics) that drive histology-agnostic treatment decisions (1). As a result, these diagnostics and drugs typically benefit small patient populations, and their clinical evaluations rarely include a randomized control group, leading to uncertainty around health and economic outcomes, both at the clinical and healthcare system level (2, 3). This uncertainty has understandably limited the reach and implementation of precision oncology technologies, with stakeholders demanding additional evidence generation mechanisms to better manage uncertainty (4–7).

In this perspective, we describe how life-cycle real-world evidence (RWE) can bridge evidentiary gaps when evaluating precision oncology technologies, including diagnostics and drugs. We define RWE as evidence that relies on purposefully generated real-world data (RWD) from healthcare systems, which includes any data collected outside the confines of trials or experiments (8, 9). The increasing reliance on expedited regulatory pathways has led to more drugs being approved on the basis of fewer or less robust clinical studies, including studies using surrogate markers as primary endpoints (10). To generate evidence not available for market approval, regulatory agencies need to rely more on life-cycle RWE for the evaluation of safety, efficacy, and comparative effectiveness (11, 12). We expand on our previously published framework for life-cycle health technology assessment (HTA), which proposes the ongoing assessment of health, economic, and societal impacts of precision oncology technologies throughout their life-cycles (3). Herein, we specify how life-cycle RWE can facilitate such assessment, reducing uncertainty about precision oncology and supporting informed healthcare decision-making. We suggest that the acceptability and validity of using RWE for supporting regulatory and reimbursement decisions require advances in methods of causal inference to inform the safety, comparative effectiveness, and cost-effectiveness of health technologies.

In the following sections, we consider how RWE can be used for regulatory and reimbursement decision-making, overviewing current RWE initiatives and identifying gaps for RWE generation and uptake. We then provide a detailed overview of common observational study biases, propose specific study designs and analytic methods for producing decision-grade RWE for precision oncology, identify real-world data system changes needed to enable these analyses, and detail how RWE can support life-cycle assessment. We conclude with a discussion of life-cycle equity analyses and RWE considerations for global healthcare systems. Box 1 defines RWE terminology used throughout this perspective (13–15).

Box 1 **Glossary of real-world evidence terminology.**

**Table:** 

**Term**	**Definition**
Bias	Degree to which the estimate is expected to be close to the true effect. A biased estimate can over- or under-estimate the true effect, potentially leading to incorrect inferences about the effect of interest.
Calendar time bias	A type of bias occurs when a synthetic control arm includes patients from different calendar times and outcomes improve over calendar time.
Causal effect estimate	A numerical value from the statistical operation used to compute the causal estimand.
Causal estimand	Mathematical expression that defines the target value to be estimated.
Causal inference	Establishing the cause and effects of the treatment or intervention being studied.
Causal model	Formal framework used to represent assumptions about the data-generating process with the goal of guiding the estimation of cause and effect.
Centralized data	Data that are managed in a unified repository facilitates access.
Confounding	A type of bias that occurs when a factor (confounder) is a common cause of both treatment and outcome, distorting the estimated causal effect. Confounders may be measured—allowing for adjustment in statistical analysis—or unmeasured.
Counterfactual identification	The process of determining whether a hypothetical scenario, e.g., what would have happened to the treated group had they not received treatment, can be answered based on a given causal model.
Decision-grade RWD	RWD that are regularly refreshed, curated, and transformed into actionable RWD, based on harmonized and documented processes, with privacy-preserving broad access to support the generation of decision-grade RWE.
Decision-grade RWE	Causal RWE that is generated from the analysis of decision-grade RWD.
Directed acyclic graphs (DAGs)	Graphical representation of a causal model with visual structures to encode assumptions about causal relationships between variables.
Health technology assessment (HTA)	A multidisciplinary process that uses explicit methods to determine the value of a health technology at different points in its life-cycle.
Life-cycle HTA	The standardization of data generation and collection, combined with methods to produce decision-grade evidence for deliberation at each phase of a technology's life cycle.
Immortal time bias	A type of bias that occurs when there is a period of follow-up time during which patients must remain outcome-free to be included in either the treatment or control arms.
Information bias	A type of bias that occurs when there is systematic measurement error in the exposure, outcome, or confounders.
Non-randomized study	A type of study in which patients are not assigned by chance to different study arms.
Observational or non- interventional study	A non-experimental study in which exposure is observed as it occurred, and includes three main study types: cohort, case–control, and cross-sectional studies.
Pragmatic trial	A type of clinical trial conducted in real-world practice designed to inform decision-making regarding the comparative balance of benefits, burdens, and risks of health interventions, based on prospectively collected RWD.
Prevalent user bias	A type of bias that occurs when patients most likely to have the outcome of interest experience early attrition and only low-risk patients are included and followed in the study.
Quasi-experimental methods	Statistical methods that are used to estimate causal relationships when randomization is not feasible or ethical. They typically rely on naturally occurring or non-randomized variations in treatment to reproduce the rigor of randomized experiments.
Random assignment emulation	An approach that mimics randomization to eliminate systematic differences with respect to known or unknown baseline characteristics between patients included in different study arms.
Real-world data (RWD)	Different sources of data collected from healthcare systems, which can include electronic medical records, clinical and disease registries, administrative databases, and any data collected outside of the confines of trials or experiments.
Real-world evidence (RWE)	Evidence on the use, safety, benefit-risk, effectiveness, and cost of health technologies that is derived from RWD.
Selection bias	A type of bias that occurs when patients included in the study are not representative of the underlying population that is the object of interest.
Siloed data	Data with limited access that are isolated within specific departments, institutions, or systems.
Standardized data	Data that follow a consistent format, structure, coding standards, and set of rules to enable integration of more than one type of RWD source captured regionally and globally across different centralized repositories.
Synthetic control arm	External control arm derived using RWD to generate a comparator for single-arm trials.
Target trial emulation	An approach to observational study design that involves first designing a hypothetical randomized trial to answer a causal research question (the “target trial”), then developing an observational (“emulation”) study mirroring this target trial. Protocols for both the target trial and emulation study must report on: study aim; eligibility criteria; treatment strategies; assignment procedures; follow-up period; outcomes; causal contrast; and statistical analysis plan.
RWD, real-world data; RWE, real-world evidence.	

### A role for real-world evidence in decision-making?

To manage evidentiary uncertainty, including for precision oncology, international regulatory, and reimbursement agencies have stated that RWE will increasingly be used in regulatory pre- and post-market decision-making and for reimbursement (16, 17). In practice, the use of RWE for regulatory market approval has been rare despite bodies such as the United States Food and Drug Administration (FDA) having a long history of using RWE to monitor and evaluate drug safety post-authorization (e.g., Sentinel System) and the European Medicines Agency (EMA) increasingly considering RWE on a case-by-case basis for regulatory purposes (18–21). Instances of regulatory approvals based on RWE have primarily been in precision oncology and rare disease settings (22). This is because single-arm trial designs are common in these settings, where small benefiting populations prohibit rapid trial accrual. In the absence of randomized controlled trial data, decision-makers require additional non-trial evidence for understanding comparators (3, 23). While the reliability of RWE is a topic of continued debate (24), several regulatory and HTA bodies support RWE backed by strong study designs and RWD for establishing safety and comparative effectiveness when randomized trials are unavailable or unable to reliably inform decision-making (25, 26). This acknowledged support signals the acceptability of RWE for causal inference, in a similar way that the satisfactory design and conduct of randomized trials can allow causal inference from a sample of people to generalize to a broader population (27, 28).

Principles for decision-grade RWE generation from non-randomized observational studies of precision oncology thus require consideration of key study design elements and methodological improvements (27, 29). Objective assessments of RWE are challenging without points of reference typical of study protocols for randomized trials (30, 31). Aiming to promote a better understanding and acceptance of RWE, regulatory and HTA bodies have been issuing study design guidance and frameworks for enabling decision-grade evidence generation. This guidance is agnostic of health technology and clinical setting, and is informative in situations in which randomized trial evidence is unavailable, when trial generalizability is of concern, or when post-market uncertainties remain. Table 1 presents RWE study design components found in selected regulatory and HTA frameworks.

**Table 1:** Study design components in frameworks for the use of real-world evidence in regulatory approval and health technology assessment.

**FDA (2024) Real-world evidence: considerations regarding non-interventional studies for drug and biological products [Draft guidance]**	**CDA-AMC, Health Canada, and INESSS (2023) Guidance for reporting real-world evidence**	**NICE (2022) NICE real-world evidence framework**	**EMA (2024) Reflection paper on the use of real-world data in non-interventional studies to generate real-world evidence**
**United States**	**Canada**	**United Kingdom**	**Europe**
*Non-interventional study design components*	*Study design reporting recommendations*	*Non-randomized study design components*	*Causal study design components*
Research question	Aim and study question	Aim	Aim
Rationale for RWE approach	Overall study design	Eligibility criteria	Eligibility criteria
Proposed approach to support causal inference	Rationale of the study design	Treatment strategies	Treatment strategies
Ethical considerations	Literature review	Assignment procedures	Assignment procedures, with attention to the prevention of:
Overall study design	Key elements of the study design (with diagrams)	Follow-up period	Selection bias
Causal diagram	A priori protocol	Outcome	Information bias
Source population	Describe study members	Causal effect of interest	Time-related bias
Eligibility criteria	Describe the study governance	Analysis plan	Confounding
Definitions for key variables	Ethics approval		Follow-up period
Relevant covariates	Funding disclosures		Outcome
Strategies to address potential bias			Causal effect of interest
Index date (time zero)			Follows estimand framework in ICH E9 (R1) guidance
Follow-up period			Analysis plan
Data sources			
Analytical approach			

FDA, United States Food and Drug Administration; CDA-AMC, Canada's Drug Agency; INESSS, Institut national d'excellence en santé et en services sociaux; NICE, National Institute for Health and Care Excellence; EMA, European Medicines Agency; ICH, International Council for Harmonization of Technical Requirements of Pharmaceuticals for Human Use.

While sharing similar aims for improving the validity and acceptability of RWE for regulatory and reimbursement deliberations, specific RWE framework components, definitions, and implementation differ across jurisdictions. For example, guidance from the National Institute for Health and Care Excellence (NICE) in the United Kingdom is prescriptive in terms of specific study design considerations for estimating causal effects of interest in non-randomized studies, requiring specification of a target trial for emulation (25). To promote understanding of study requirements to generate causal evidence of effectiveness and/or evidence of safety, the FDA recently updated draft guidance recommendations for non-interventional study designs, which mandate similarly transparent reporting of study design considerations, but do not explicitly require targeted trial emulation (16, 26). Canada's Drug Agency (CDA-AMC), Health Canada, and the Institut national d'excellence en santé et en services sociaux (INESSS) collaborated on a reporting framework to assist appraisal of RWE study components (32). CDA-AMC also released methods guidance for HTA that highlights RWE quality appraisal considerations and, similar to the FDA, suggests modern causal inference frameworks such as target trial emulation, but makes no specific methodological recommendations (32, 33). The EMA's recent draft reflection paper on key methodological considerations for non-interventional studies to generate RWE for regulatory purposes draws from both existing and in-development frameworks of the International Council for Harmonization of Technical Requirements for Pharmaceuticals for Human Use (ICH) (34–36). In an effort to align RWE policies across Asia, a collaboration of academics and HTA agencies in the region, the REAL World Data In ASia for HEalth Technology Assessment in Reimbursement (REALISE) working group, developed non-binding recommendations that include specific guidance on statistical methods but do not recommend any specific study design (37, 38). Calls remain to establish methodological standards for RWE studies (35, 39–41), and the FDA continues to oversee and support demonstration research projects to determine study designs that can generate substantial evidence for regulatory decisions (26, 42).

We contend that harmonization across regulatory and HTA bodies on study design components will promote a better understanding and acceptance of RWE and ultimately form the backbone of agile regulation environments adapted to rapid healthcare innovation, which is inherent to precision oncology. Harmonization efforts must be centered on accepted methodological approaches and study designs that mitigate potential sources of bias to reliably inform regulatory and reimbursement decision-making.

### What is the real-world evidence study design and method for analysis?

Skepticism about using RWE in regulatory and reimbursement decisions primarily arises from concerns about biases common in observational studies, but absent in well-conducted randomized controlled trials (24, 43). In particular, confounding bias occurs when a patient characteristic or external factor is associated with both the mechanism of treatment assignment and the outcome of interest. For example, in the case of precision oncology, a key confounder may be the timing of an ‘omics-based diagnostic or treatment relative to cancer diagnosis (44). Failure to adjust for this confounding obfuscates the estimated effect of treatment, resulting in the inability to conclude causality of the effect. The act of randomization eliminates confounding bias by producing a treatment and control group that is well balanced in both observable and unobservable characteristics, including genetic factors. This balance ensures that any measured change in outcomes for the treatment group is attributable to treatment itself rather than systematic differences across the two groups studied.

To avoid confounding bias and ensure that RWE can be reliably used for precision oncology decision-making, we contend that RWE generation must be grounded within a causal theoretical framework. The framework should make explicit the complex, intersecting relationships between observed and unobserved variables and the estimand of interest. Based on the theory underlying Structural Causal Modeling, directed acyclic graphs (DAGs) are a graphical tool for transparently mapping these inter-variable relationships (45). DAGs are a non-parametric, diagrammatic means of representing the complete set of variables involved in a data generating process, that use nodes and unidirectional arcs to depict possible causal and non-causal relationships (46). Included variables and their theorized relationships should mirror real-world patient experiences and can be based on systematic evidence review, behavioral models, large-language models, and stakeholder consultation (47, 48). DAGs enable the manual or algorithmic determination of minimally sufficient covariate adjustment sets for comparative evaluations (49–51). Adjusting for only those covariates included in a sufficient adjustment set guarantees we are able to answer our causal question, without blocking causal paths or unnecessarily reducing degrees of freedom, better powering precision oncology evaluations in rare indications.

### Analytical approaches for precision oncology applications

In non-randomized, single-arm precision oncology settings, quasi-experimental approaches can be used alongside causal theoretical frameworks to reliably support regulatory and reimbursement deliberations (50). Quasi-experimental approaches, such as matching or inverse probability of treatment weighting, appear in HTA evidence packages, particularly for new drug classes such as tumor-agnostic treatments and orphan drugs (52, 53). These approaches can enable counterfactual identification of single-arm precision oncology trial participants while addressing both observed and unobserved sources of confounding occurring before and/or after study inclusion. The appropriate quasi-experimental approach depends on the research question, clinical context, primary endpoints, sources of confounding, and available data. Careful consideration of strengths, limitations, and underlying assumptions for competing methods is necessary for unbiased and statistically efficient estimation. For example, matching emulates the effects of randomization on cohort characteristics and enables estimation of an average treatment effect on the treated (ATT) population, but also reduces available data and can be problematic to apply in finite samples common to precision oncology, where genomic heterogeneity results in small benefiting populations (54). Inverse probability of treatment weighting instead estimates an average treatment effect (ATE) using all available data and performs relatively well in rare outcome settings (55), but is sensitive to model misspecification and extreme weights. Both approaches assume positivity and ignorability, requiring thorough overlap and balance assessments to confirm validity.

Once a quasi-experimental approach is selected, the study must be carefully designed to avoid self-inflicted biases (30, 56). Study criteria, exposure, and follow-up definitions each have implications for time and selection-related biases that must be considered alongside analytical correction methods. For example, defining study eligibility and initiating follow-up after receipt of a precision oncology intervention may lead to both selection bias and prevalent user bias (56, 57). Selection bias occurs when study eligibility is non-random, resulting in a study cohort that no longer represents the target population of interest (58). Prevalent-user bias occurs when patients most likely to experience the outcome of interest, such as disease progression or death, experience attrition, and follow-up is limited to only low-risk patients (59). Even study calendar time can introduce bias if differential access to care occurring across treated and control patients remains unaccounted for (60).

For progressive diseases such as cancer, patient prognosis, and treatment landscapes change continuously over time, and study indexing and follow-up definitions are especially important. If the study design allows for delayed entry, where follow-up is initiated after the natural time of origin for some but not all patients, time-varying characteristics, and prognosis may be systematically different from at the time of origin, introducing confounding that must be adjusted (61, 62). Immortal time bias is related to delayed entry and occurs when, by design, there is a period of follow-up time during which either treated or control patients must remain outcome-free (63). For example, if patients experience a delay in accessing ‘omics-based testing after diagnosis, or in receiving targeted treatment after biomarker results (64). While immortal time bias remains common in oncology evaluations (65, 66), this bias can be mitigated through immortal time adjustment methods, such as time-dependent analysis, multiple imputation, participant cloning, or the use of a prevalent new-user cohort design (60, 64, 67). Best practice guidelines on RWE study design and analysis are critically needed to avoid biases common to precision oncology evaluations and practically support decision-grade evidence generation throughout health product life-cycles.

### What are real-world data and data systems?

DAGs outline the minimum set of RWD elements needed for causal inference on how a precision oncology intervention affects clinical, economic, and health system outcomes. DAG-informed data elements may include a mix of historical and prospectively collected data, necessitating longitudinal integration of genomic profiling information with other health data such as patient socio-demographic characteristics, disease characteristics, patient-reported outcomes, and clinical outcomes such as overall and progression-free survival (48, 68). Additionally, retrospective and future data on resource use and cost of genomic profiling, prescribed treatment, in-patient hospital stays, physician visits, non-genomic laboratory tests, clinical outcomes, adverse events, and non-cancer prescription drugs are important for valid causal inference on health outcomes and for estimating downstream cost-effectiveness, respectively.

While past efforts have defined core RWD elements needed for precision oncology evaluations (44, 68), the ability of healthcare systems to curate the required data is variable. Missing elements threaten the generalizability and validity of RWE outcomes analyses, with missing data points, measurement errors, and data misclassifications contributing to information bias (58, 69). Data curation is the organization and integration of data collected from siloed sources (70, 71). RWD curation involves the annotation and maintenance of data over time, and may require abstracting important text, image, and other health services information from a variety of structured and unstructured sources, such as electronic medical records, administrative data, registries, patient-reported surveys, or social media (9). As such, data curation incorporates a range of activities and processes, which include cleaning and normalizing data, adding metadata and quality verification, all necessary to create, manage, maintain, and validate required data elements, grounded in a common data model (70, 71). These standardization efforts are critical for achieving complete capture of precision oncology core data elements, facilitating data sharing across institutions and jurisdictions to not only increase generalizability but also augment available sample sizes in rare indications (72).

RWD curation, sharing, and use in precision oncology decision-making remain limited in practice. Healthcare systems face legal, structural, and operational barriers. For example, the generation and use of RWD may be impeded by data stewards' interpretation and implementation of legislation that governs privacy and health information, resulting in persistent data siloing across institutions and jurisdictions (72–74). Systematic use of RWD has been largely confined to individual clinical decision-making, hospital performance reporting, and quality improvement (8, 75, 76). Addressing these barriers while generating regularly refreshed, curated, and transformed actionable data based on harmonized and documented processes is necessary for generalizable decision-grade RWD. Broad privacy-preserving access to decision-grade RWD can then support the generation of decision-grade RWE, ultimately enabling real-world life-cycle assessment.

### Real-world life-cycle assessment

**Figure 1:**
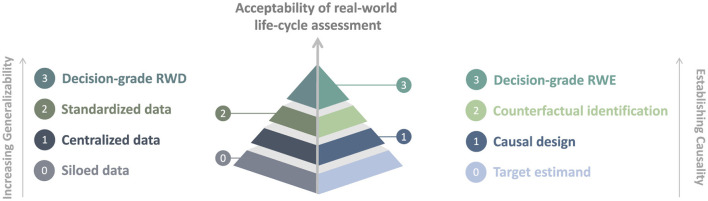
Hierarchy for the acceptability of real-world life-cycle assessment.

Life-cycle activities have always been part of the market approval and reimbursement process (12, 77); however, there remains a need for greater transparency and guidance on the implementation of life-cycle assessment (78, 79). Figure 1 illustrates a hierarchy for the acceptability of real-world life-cycle assessment that builds on previous sections detailing the needs for the harmonization of RWD and data systems and of causal RWE approaches.

Life-cycle assessment of outcomes can be used both in regulatory and reimbursement decisions as well as for re-appraisal and disinvestment (3). For example, prior to market authorization and health system reimbursement, a precision oncology intervention may be eligible for managed access, which could support conditional access to technologies whose comparative outcomes are uncertain. To create a managed access agreement, the healthcare system and the sponsor of the health product would agree on research that uses historical data alongside prospectively collected clinical trial and healthcare system data. The RWD would align to the DAG-identified minimum adjustment set and to a core data set for the cost-effectiveness of precision oncology. Broad initiatives for operationalizing RWD for causal analysis are showing promise (48). RWE generated can inform the continuation of managed access, as well as address any post-market conditions of regulatory authorization. Using an evidence-based definition to determine when substantial evidence exists based on life-cycle RWE would also promote transparency and accountability for both stakeholders and regulators (80). In the post-reimbursement phase, continual outcomes surveillance and health technology management provide the opportunity for comparative evaluation of precision oncology interventions versus alternative historical or contemporaneous standards of care, using quasi-experimental cohort comparisons. The outcomes of these phases can inform disinvestment in low-value technologies, freeing resources for other areas of innovation or healthcare.

A scoping review of regulatory and HTA policies recently underscored the need for better alignment on the use of RWE methods (40, 81, 82). Greater clarity and standardization of guidelines can help leverage RWD for improving healthcare decisions (39, 48). Similar needs have been highlighted in the context of international collaboration on RWD and RWE for regulatory decision-making (41). A real-world life-cycle assessment approach with transparent decision-making that limits duplicative efforts for evidence generation in precision oncology can provide meaningful value in addressing the decision problems that expedited regulatory pathways aim to resolve.

## Discussion

Real-world life-cycle assessment is a promising solution for addressing precision oncology uncertainty. While regulators and reimbursement agencies are receptive to RWE in settings where randomized controlled trials are limited or unavailable, RWE continues to be infrequently used in decision-making. Routine, health system-integrated life-cycle assessment of precision oncology diagnostics and treatments remains elusive. Stakeholder-driven data and methods guidance are needed to achieve consensus on acceptable forms of RWE, promote evidence generation, and ensure uptake. This guidance should be anchored in the study design and analytical principles discussed throughout this perspective. Any RWE used for regulatory and reimbursement purposes should adhere to an underlying causal theoretical framework, with carefully justified and validated study designs and model estimation that mitigate biases common to observational precision oncology evaluations. While there is emerging consensus found in RWE guidance frameworks supporting causal designs based on target trial emulation (25, 26, 32, 33), its use in submissions is still limited (83). Specific recommendations on important drivers of uncertainty in precision oncology evaluations, such as accurately defining index dates and approaches to emulate randomization for external control arms (84–86), remain necessary to promote the use of real-world trial emulation (82, 83, 87). Supporting RWD curation must meet core precision oncology data requirements for enabling causal analyses, while harmonizing to allow for cross-jurisdictional RWD sharing. Combined, these efforts can facilitate real-world life-cycle assessment that drives sustainable resource allocation for precision oncology.

To simultaneously achieve equitable precision oncology implementation, equity analyses should be considered and embedded in every phase of the life-cycle assessment. During early product prioritization, RWE characterizing differential condition prevalence, access to care, and opportunity to benefit can ensure that research and development explicitly considers the priority population and avoids creating or exacerbating inequities (88, 89). This evidence is particularly salient for precision oncology, in which both drug development and patient selection for treatment depend on the use of genomic variant background libraries that historically underrepresent populations of non-European ancestry (90, 91). Following product development and evaluation, distributional analyses of comparative effectiveness and cost-effectiveness can provide regulators and reimbursement agencies with evidence of precision oncology outcomes variation, including for equity-seeking groups (2, 92, 93). This evidence can inform implementation strategies that mitigate disparities, supported by post-market RWE monitoring of access and uptake across diverse populations.

To fully realize the potential of life-cycle RWE for achieving equitable healthcare, careful considerations of global health system factors will be crucial to future research and operations initiatives. Health systems will vary in their ability to effectuate the life-cycle framework described herein, with reliable real-world assessments of precision oncology interventions requiring substantial investment in RWD infrastructure alongside legal and regulatory modernization. Both financial and non-financial resources will be necessary to support continuous RWE generation and integration into decision-making. Low- and middle-income countries face competing challenges for cancer control, such as public awareness and health literacy, healthcare funding and infrastructure, oncology workforce capacity, cancer screening availability and uptake, diagnostic turnaround times, treatment abandonment, and a lack of palliative care (94). High costs of precision oncology diagnostics and drugs are prohibitive in constrained resource settings, where feasible, high-impact, cost-effective interventions are prioritized (95–97). Federated analytics and validation of RWE transportability across jurisdictions present near-term opportunities for translating life-cycle assessment into global health systems (9, 97). These solutions require thoughtful, partnered design, validation, adjustment, and regulation to ensure suitability for all contexts (98, 99).

## Conclusion

Uncertain comparative evidence for ‘omics-based diagnostics and treatments challenges regulatory and reimbursement decision-making globally. As a result, precision oncology implementation remains limited to select patient populations in a few jurisdictions. Real-world life-cycle assessment can reduce evidentiary uncertainty, supporting the sustainable and equitable implementation of precision oncology across healthcare systems. Stakeholder-driven guidance on acceptable RWE methods for unbiased causal inference and significant health data system augmentation is, however, required to achieve a life-cycle assessment. In the long run, global partnerships that leverage innovative, diagonal financing mechanisms alongside health system strengthening may be necessary to deploy real-world life-cycle assessment and bridge the evidentiary gaps in precision oncology worldwide.

## Data Availability

The original contributions presented in the study are included in the article/supplementary material, further inquiries can be directed to the corresponding author.
